# Timed saliva sampling as a new reliable marker for assessing iodine intake

**DOI:** 10.2478/raon-2026-0045

**Published:** 2026-09-07

**Authors:** Adrijana Oblak, Jernej Imperl, Mitja Kolar, Gregor Marolt, Blaz Krhin, Katja Zaletel, Simona Gaberscek

**Affiliations:** 1 Division of Nuclear Medicine, University Medical Centre Ljubljana, Ljubljana, Slovenia; 2 Faculty of Medicine, University of Ljubljana, Ljubljana, Slovenia; 3 Faculty of Chemistry and Chemical Technology, University of Ljubljana, Ljubljana, Slovenia

**Keywords:** Sandell-Kolthoff reaction, salivary iodine concentration, iodine excretion in the 24-hour urine, ICP-MS, saliva, iodine intake, iodine concentration

## Abstract

**Background:**

Iodine is an essential component of thyroid hormones. Therefore, sufficient iodine intake is important for adequate thyroid function. Measuring the urinary iodine concentration (UIC) is the recommended method for assessing population iodine intake, but it has certain drawbacks. In addition to urine, iodine is also excreted into saliva. Therefore, the aim of our work was to determine whether salivary iodine concentration (SLIC) measurement could replace UIC measurements, even a gold standard, urinary iodine excretion (UIE) in the 24-hour urine. We also evaluated the influence of saliva collection time on SLIC measurements.

**Subjects and methods:**

We invited adult volunteers who first provided 24-hour urine and saliva samples collected on the same day at home, strictly according to the protocol. UIC and SLIC before and after meals were measured using the Sandell-Kolthoff method, comparable to inductively coupled plasma mass spectrometry.

**Results:**

The study included 104 healthy participants from whom all biological samples were completely collected. Importantly, none of them was taking thyroid medication or iodine supplements and all were without thyroid disease. A very good correlation of SLIC values at 60 and 120 min after the first and second meal and 30 min after the second, third, and fourth meals with UIE was observed (P>0.05).

**Conclusions:**

We found an excellent correlation between SLIC values and UIE, where the SLIC value depends on the sampling timing and/or number of meals. SLIC measurement could replace UIE measurement and represents an important and possible new biomarker for assessing iodine intake.

## Introduction

Approximately one-third of the world’s population does not consume enough iodine^1^, leading to an increased risk of developing iodine deficiency disorders^2^ and a higher risk of thyroid disease.^3^ With adequate iodine intake, people with healthy thyroid glands excrete approximately 90% of the ingested iodine in their urine.^2^ Therefore, measurement of urinary iodine concentration (UIC) is recommended to assess iodine intake. UIC can be measured from a single urine sample or from a 24-hour urine collection, assessing daily urinary iodine excretion (UIE), the latter being considered the gold standard.^1^ Consequently, most population-based studies have used urine as the primary sample matrix for the assessment of iodine intake. However, owing to the drawbacks of urine sampling, such as fluid intake, diuresis, dietary factors, and (patho)physiological factors (pregnancy, renal function)^4,5^, UIC and UIE measurements should be interpreted with caution, especially at the individual level. In light of these limitations, the presence of sodium iodide symporter molecules in the salivary glands^6^, which enables iodine excretion into saliva, highlights saliva as a promising alternative sample for the assessment of iodine intake.

Several methods have been developed to determine iodine content.^7^ In common practice, we normally use two methods for iodine measurement in biological samples: method using the Sandell-Kolthoff (S-K) reaction with various pretreatment steps and inductively coupled plasma mass spectrometry (ICP-MS).^8^ While the S-K method is easier to handle and less expensive than ICPMS, owing to the toxic effect of arsenic, which is used in the S-K reaction, careful waste disposal is required.^9^ On the other hand, although the ICPMS method is more accurate, it is more complex to handle and requires more trained personnel. The S-K method is also suitable for the measurement of salivary iodine concentration (SLIC) and yields results comparable to those obtained using ICP-MS.^10^ In addition, thiocyanate, present in the saliva of tobacco smokers^11^, and caffeine, which is abundant in caffeinated drinks, do not interfere with SLIC measurements. Previous studies on schoolchildren have shown that the amount of iodine in saliva increases during the course of the day.^12^

The aim of our work was to determine whether SLIC measurement could replace UIE measurement in the 24-hour urine collection to assess population iodine intake. Furthermore, we also wanted to establish a correct time window for saliva collection to measure the SLIC.

## Subjects and methods

### Study design and institutional review board approval

This cross-sectional study was conducted between May 2022 and February 2023. One hundred and forty-five adult participants from the local population were invited to participate in the study, approved by the Slovenian National Medical Ethics Committee (identification number 0120-271/2021/3). The study was completed in accordance with the Declaration of Helsinki as revised in 2013. All the participants provided informed consent. Only participants without known thyroid diseases were included in the study. The exclusion criteria were: presence of thyroid antibodies, failure at providing of all saliva and urine samples, consuming iodine-containing supplements or water treated with iodine. Blood samples were collected from each participant on the day of the study. After clotting, the samples were centrifuged at 1800 x G for 10 min at room temperature, followed by thyroid-stimulating hormone (TSH), thyroglobulin antibodies (anti-Tg), and thyroid peroxidase antibodies (anti-TPO) measurement. On the same day, all participants collected 24-hour urine samples and all saliva samples before (0 min) and 30, 60, and 120 min after each meal, regardless of the time and number of meals consumed. For the 24-hour urine sample collection, 3.3-liter bottles (Becton Dickinson, USA) were used. Subsequently, two urine samples were aliquoted using Vacuette® (10mL, Greiner Bio One, GmbH, Austria) for UIC and creatinine determination, and the volume of urine collected was determined. Salivettes® (Sarstedt, Germany) were used for the saliva samples. Each sample was stored in a freezer and transported to the laboratory within 24 hours. The saliva samples were centrifuged at 1800 x G for 10 min at room temperature, and all urine and saliva samples were frozen at -80°C before analysis.

The participants completed a questionnaire in which they stated their smoking status, intake of dietary supplements and medication, and use of (non-)iodized salt.

### Methods

TSH, anti-Tg and anti-TPO levels were measured using Atellica® IM 1600 Solution according to the manufacturer’s instructions (Siemens Healthineers, Germany). TSH was measured using the TSH3-Ultra assay, with intra-and inter-assay coefficients of variation (CV) ranging from 1.2%–3.6% and 2.6%–4.5%, respectively. The TSH reference interval for the adult population was defined by the laboratory.^13^ Anti-Tg was measured using the analyte-bridging immunoassay aTgII, with intra-and inter-assay CV ranging from 1.5%–2.5% and 2.0%–3.7%, respectively. Anti-TPO was measured using the competitive immunoassay aTPO, with intra-and inter-assay CV ranging from 1.5%–4.1% and 3.4%–7.4%, respectively. Urinary creatinine was measured using the Dimension Vista® 1500 Inteligent Lab System (Siemens Healthineers, Germany) using enzymatic quantitative creatinine determination. The intra-and inter-assay CV ranged from 0.8%–2.9% and 1.7%–4.4%, respectively.

UIC and SLIC were measured by the spectrophotometric method on microplates based on the S-K reaction with ammonium peroxydisulfate digestion of samples prior to analysis, both comparable to ICP-MS.^10,14^ The S-K reaction utilizes the catalytic activity of iodide ions in the reaction of arsorous acid and ammonium cerium (IV) sulfate in sulfuric acid solution. The presence of iodide increases the reaction rate, which is directly proportional to the concentration of iodide in the solution.^15^ For UIC, the intra-and inter-assay CV were 2.3%–2.5% and 4.9%–6.3%, respectively^14^, while for SLIC, the intra-and inter-assay CV were 2.8%–18.4% and 4.3%–20.7%, respectively.^10^ In brief, 1 mL of 1 mol/L ammonium peroxydisulfate solution was added to 250 μL of the standards, controls, and samples. The solution was mixed and incubated for 1 h at 95°C in a dry bath. After cooling to room temperature, 50 μL of the standards, controls, and samples were transferred in duplicates to the microplate. An arsorous acid solution (100 μL) was added to each well. The microplate was then sealed and incubated for 60 s on a microplate shaker. Subsequently, 50 μL of ammonium cerium (IV) sulfate solution was added to each well within 40 s. The microplate was sealed and incubated on the microplate shaker for exactly 30 min. Immediately after shaking, the absorbance was measured at 405 nm using a spectrophotometer (SunriseTM, Tecan, Switzerland).

The Kolmogorov-Smirnov test was used to assess the normality of the data distribution. None of the data were normally distributed; therefore, all data are presented as medians with interquartile ranges (IQR), except for age, which is presented as the mean and associated range. Inferential statistics were performed using the Mann-Whitney test. Statistical analysis was performed using MedCalc Statistical Software version 22.021 (MedCalc Software bvba, Ostend, Belgium) with statistical significance set at P < 0.05.

## Results

In total, 145 participants were included in this study. Three participants declined participation in the study, 20 participants had antibodies against anti-TPO and/or anti-Tg and were later removed from the study. Fifteen participants failed to provide all saliva and/or urine samples, and 3 participants consumed iodine-containing supplements and were therefore excluded from the analysis. The characteristics of the study population are summarized in Table 1.

**TABLE 1.** Participant characteristics

Demographic data		N = 104
Age (y) (range)		43 (23-74)
Gender (%)	Men	32 (30.8)
	Women	72 (69.2)
TSH (mIU/L) (IQR)		1.68 (1.22-2.30)
Smoking (%)	Smokers	7 (6.7)
	Non-smokers	97 (93.3)
Salt (%)	Iodized	98 (94.2)
	Non-iodized	6 (5.8)

IQR = interquartile range; TSH = thyroid-stimulating hormone

The results of all measurements and calculations for iodine and creatinine in 24-hour urine are shown in Table 2. All 24-hour urine samples had a creatinine concentration above 0.2 g/L and volume of urine above 0.5 L and were thus suitable for further measurements.

**TABLE 2.** Median volume, measured and calculated values of iodine and creatinine in 24-hour urine

N = 104	Median (IQR)
Volume of 24-hour urine (L)	2.0 (1.4-2.7)
Measured iodine concentration in 24-hour urine (μg/L) - UIC	70.6 (50.7-95.2)
Measured creatinine concentration in 24-hour urine (g/L)	0.61 (0.47-0.86)
Calculated amount of iodine in 24-hour urine (μg) – UIE	137.1 (103.3-173.2)
Calculated amount of creatinine in 24-hour urine (g)	1.18 (0.98-1.59)
Calculated amount of iodine/creatinine in 24-hour urine (μg/g)	108.6 (82.5-134.0)

IQR = interquartile range; UIC = urinary iodine concentration; UIE = urinary iodine excretion

Participants consumed 2–4 meals per day, with 2 meals consumed by 12/104 (11.5%) participants, 3 meals consumed by 66/104 (63.5%) and 4 meals consumed by 26/104 (25.0%) participants. The results of the individual SLIC medians per meal are shown in Table 3.

**TABLE 3.** Median SLIC per meal, including different time points of saliva sampling, before (0 min) and 30, 60, and 120 min after the meal. The median SLIC was also compared among the different time points of saliva sampling, and a comparison was made with UIE

Meal	N	Time of sampling (min)		SLIC (μg/L)	^1^P	^2^P	^3^P	^4^P	^5^P	^6^P
1	104	0	median (IQR)	92.7 (60.2-166.1)	P = 0.994	P = 0.208	P = 0.013	P = 0.005	P = 0.002	P = 0.001
		30		100.3 (73.3-136.4)						P < 0.001
		60		119.7 (90.7-168.9)	P = 0.533					P = 0.138
		120		123.2 (90.9-178.7)						P = 0.491
2	104	0	median (IQR)	76.7 (53.7-115.3)	P < 0.001	P < 0.001	P < 0.001	P = 0.003	P = 0.006	P < 0.001
		30		110.0 (83.7-182.6)						P = 0.064
		60		141.1 (102.4-237.5)	P = 0.925					P = 0.176
		120		159.9 (87.9-235.5)						P = 0.116
3	92	0	median (IQR)	98.3 (62.5-146.7)	P = 0.001	P < 0.001	P < 0.001	P = 0.007	P =0.002	P< 0.001
		30		127.3 (97.1-183.7)						P = 0.981
		60		163.0 (120.6-276.5)	P = 0.590					P = 0.002
		120		163.0 (120.6-276.5)						P < 0.001
4	26	0	median (IQR)	108.2 (66.1-163.6)	P = 0.464	P = 0.001	P = 0.002	P = 0.006	P = 0.004	P=0.040
		30		112.2 (98.9-154.2						P = 0.163
		60		173.8 (136.1-237.9)	P = 0.714					P = 0.012
		120		183.8 (120.9-268.6)						P = 0.012

1 P-value, comparison of SLIC before (0) meal and 30 min (above) after, and 60, and 120 min (below) after meal,

2 P-value, comparison of SLIC before (0) meal and 60 min after meal,

3 P-value, comparison of SLIC before (0) meal and 120 min after meal,

4 P-value, comparison of SLIC 30 and 60 min after meal,

5 P-value, comparison of SLIC 30 and 120 min after meal,

6 P-value, comparison of SLIC before (0) meal and after meal (30 min, 60 min and 120 min, respectively) with UIE.

IQR = interquartile range; SLIC = salivary iodine concentration; UIE = urinary iodine excretion

Looking at the first meal, the results showed no statistical difference in the SLIC values between before (0 min) and 30 and 60 min after the meal. A statistical difference was found between 0 and 120 min after the meal. Additionally, there were also statistical differences in the SLIC values between 30 and 60 min after the meal and between 30 and 120 min after the meal. In contrast, there were no statistical differences in the SLIC values between 60 and 120 min after the meal. For the second and third meals, there was a statistical difference in SLIC values before the meal and at all other times after the meal, whereas there was no difference in SLIC values between 60 and 120 min after the meal. At the fourth meal, there was no statistical difference in the SLIC values between the pre-meal and 30 min post-meal measurements or between the 60-and 120-minute post-meal measurements. However, comparisons among post-meal time points showed a statistical difference in the SLIC values. After comparing all SLIC values at different times with UIE, the SLIC values at 60 and 120 min after the first and second meal and 30 min after the second, third, and fourth meals were comparable with UIE (P > 0.05), while all other values were not comparable (P < 0.05).

The comparison of the SLIC values before the meal (0 min) showed that there were no statistically significant differences between the first and third meal and between the first and fourth meal (P = 0.860 and P = 0.942, respectively), and the same applies to the second and fourth meals and between the third and fourth meals (P = 0.106 and P = 0.884, respectively). At the same time, statistical differences were observed between the first and second meals and between the second and third meals (P = 0.008 and P = 0.014, respectively). Similarly, comparison of all 120-minute post-meal SLIC values showed no statistical differences between the first and second, between the second and third, between the second and fourth and between the third and fourth meals (P = 0.081, P = 0.125, P = 0.200, P = 0.974, respectively), but there was a statistical difference between the first and third meals and between the first and fourth meals (P < 0.001 and P = 0.009, respectively).

SLIC values increased with number of meals. The median SLIC value increased after each meal, regardless of meal time. In addition, the median SLIC value also increased with the number of meals per day and was the highest 120 min after the fourth meal. Importantly, a statistical difference in SLIC values was also observed 120 min after each meal and immediately before the next meal (i.e., 0 min), namely, between the first and second meal (P < 0.001), between the second and third meal (P < 0.001), and between the third and fourth meal (P = 0.004). Additionally, SLIC values were compared according to the number of meals consumed. There was a statistically significant difference between the first meal and 120 min after the last meal if a participant had consumed 2 meals/day (P = 0.002), three meals/day (P < 0.001) or four meals/day (P < 0.001). In addition, there was also a statistical difference between participants who ate three or four meals/day (P = 0.001).

## Discussion

In recent years, some studies have assessed the suitability of SLIC for iodine intake estimation.^12,16^ To our knowledge, this is the first study to compare excreted iodine in saliva before and at different times after each meal with UIE to determine the dynamics of iodine excretion into saliva. Our research showed an important breakthrough in this field, namely, an excellent correlation between the SLIC value and UIE. The results showed that the SLIC value at 60 min and 120 min after the first and second meal, as well as SLIC value 30 min after the second, third, and fourth meal, is comparable to UIE, which is currently the gold standard method and is therefore suitable for assessing iodine intake.

Adequate iodine intake is essential for normal thyroid function. This is usually achieved by adequate iodization of table salts.^17^ However, in some countries, this can also be achieved through dietary habits.^18^ The WHO recommends assessing iodine intake at the population level by measuring UIC in a single urine sample; however, the timing of urine collection is not suggested.^1^ UIC measurements should be interpreted with caution, considering the influence of diuresis (urine sample dilution effect) and the timing of urine sample collection^19^, especially at the individual level. As iodine is also excreted in saliva, this biological sample offers an additional option for assessing iodine intake. Among 104 adults, 32 (30.8%) men and 72 (69.2%) women participated in our study. As already shown, there are no differences in gender in UIC determination^19^; therefore, gender should not influence the SLIC values. Most (94.2%) participants consumed iodized salt or at least a combination of iodized/non-iodized salt, which is consistent with research in our country, where only limited non-iodized salt is available or used.^20^

The gold standard method for assessing population iodine intake is currently UIE, which is why all SLIC measurements were compared with it. Creatinine concentration was measured in all urine samples to determine the quality of the sample and to reduce the possible impact of diuresis on UIC results. The median UIC value in 24-hour urine was 70.6 μg/L. However, this value indicates little about the daily iodine intake in our participant group, as this value also depends on the fluid intake and volume of collected urine. Considering the median volume of 24-hour collected urine, which was 2.0 L and corresponds to normal urine excretion per day^21^, the calculation of UIE yields a median value 137.1 μg iodine/day. It is known from the literature that in healthy people, 90% of the daily iodine intake is excreted via urine.^2^ Accordingly, the median iodine intake of our group was approximately 150 μg iodine/day, which corresponds to WHO recommendations.^1^

Due to the disadvantage of time-consuming collection and the possibility of improper sampling of 24-hour urine^22^, our aim was to evaluate the results of SLIC at different individual time points of saliva sampling with UIE in healthy individuals.

The comparison of SLIC values at different sampling times before/after a meal and independent of the number of meals per day showed that SLIC values between pre-meal and 30 min after a meal were statistically different from SLIC values between 60 and 120 min after a meal (P < 0.05), with only one exception, the first meal, where there was no difference in SLIC value before and 60 min after the meal (P = 0.208). Interestingly, there are statistical differences in the SLIC before and 30 min after the second and third meals, but not after the first and fourth meals. This could be due to a time lag between iodine absorption via the intestine and iodine transfer to the salivary glands during the first half of the day. According to literature data, iodine is excreted into saliva approximately 10–15 min after food intake^23^, and SLIC increases with time after all meals. The increased salivary excretion (along with iodine) after a meal may be related to antimicrobial protection of the oral and gastric mucosa.^24,25^ Importantly, there was a statistically significant difference in SLIC levels after (120 min) a meal and before (0 min) the next meal (P < 0.05). The reason for this could be that iodine requirements and its effect on the oral cavity decrease with time after food intake (at least 120 min). Interestingly, the SLIC increased after each meal throughout the day, and not only after each meal. The highest SLIC was observed in the evening. Similar findings have been reported in children.^12^ Notably, the results showed that SLIC levels at 60 and 120 min after the first and second meals, as well as 30 min after the second meal, were comparable to UIE. Therefore, saliva could replace iodine measurement in 24-hour urine, which is very difficult to collect, especially for elderly individuals.^22^ Furthermore, SLIC values at 30 min after the third and fourth meals were comparable to those of excreted iodine in 24-hour urine. This could be consistent with the increase in iodine concentration throughout the day, which could lead to a more rapid increase in SLIC levels when healthy individuals consume more than 2 meals per day. The advantage of saliva sampling is that it is a faster and simpler protocol for assessing iodine intake, allowing sampling at any point in time, regardless of the number of meals per day. The results showed that the timing of saliva sampling could be related to meals and not to a specific time of day. In addition, individuals can collect saliva sample at home/work at their own pace and send the sample to the laboratory instead of traveling to a medical facility. Only a small group of smokers (7) participated in our study and were included in the analysis because thiocyanate and coloured saliva, which are present in smokers, do not affect SLIC measurements using the S-K method.^10^

The advantage of our work was that the collection of urine and saliva sampling took place on the same day in the participants’ own environment, which allowed simultaneous monitoring of iodine excretion in saliva and the 24-hour collected urine. In addition, all participants taking dietary supplements that affect iodine measurements were excluded from calculations. More than 100 participants took part in this study. A disadvantage of saliva sampling could be the poor hydration of the individual, which could lead to lower saliva volume and consequently higher SLIC values^26^, especially in the elderly.^27^ This problem could be solved by using constantly secreted analytes in saliva as an internal standard, such as amylase^28^, which is also used in forensics.^29^

For the additional support of the research, it would be important to extend the study to population-based groups of people to confirm the data at a national level, and to study groups of people with known thyroid disease, as well as those who deliberately avoid iodized salt or iodine intake. It would also be important to investigate any possible interferences affecting SLIC measurements, particularly in participants taking medication, determine relevant cut-offs for SLIC, and, to assess inter-and intraindividual variability of SLIC.
